# Get2PrEP2—A Provider Messaging Strategy to Improve PrEP Uptake: A Randomized Controlled Quality Improvement Project

**DOI:** 10.1093/ofid/ofae297

**Published:** 2024-06-20

**Authors:** Jason Zucker, Delivette Castor, Deborah Theodore, Caroline Carnevale, Eshiemomoh Osilama, Norman Archer, Lily Bonadonna, Elena Wadden, Nicholas Morley, Simian Huang, Kathrine Meyers, Alwyn Cohall, Peter Gordon, Magdalena E Sobieszczyk

**Affiliations:** Department of Medicine, Division of Infectious Diseases, Columbia University Irving Medical Center, New York, New York, USA; HIV Prevention Program in the Comprehensive Health Program, Ambulatory Care Network West, NewYork-Presbyterian Hospital, New York, New York, USA; Columbia University Vagelos College of Physicians and Surgeons, New York, New York, USA; Department of Medicine, Division of Infectious Diseases, Columbia University Irving Medical Center, New York, New York, USA; Columbia University Vagelos College of Physicians and Surgeons, New York, New York, USA; Department of Epidemiology, Mailman School of Public Health, New York, New York, USA; Department of Medicine, Division of Infectious Diseases, Columbia University Irving Medical Center, New York, New York, USA; HIV Prevention Program in the Comprehensive Health Program, Ambulatory Care Network West, NewYork-Presbyterian Hospital, New York, New York, USA; Columbia University Vagelos College of Physicians and Surgeons, New York, New York, USA; HIV Prevention Program in the Comprehensive Health Program, Ambulatory Care Network West, NewYork-Presbyterian Hospital, New York, New York, USA; Department of Medicine, Division of Infectious Diseases, Columbia University Irving Medical Center, New York, New York, USA; Geisinger Commonwealth School of Medicine, Scranton, Pennsylvania, USA; Department of Medicine, Division of Infectious Diseases, Columbia University Irving Medical Center, New York, New York, USA; UCSF School of Medicine, San Francisco, California, USA; Department of Medicine, Division of Infectious Diseases, Columbia University Irving Medical Center, New York, New York, USA; Wayne State University School of Medicine, Detroit, Michigan, USA; Columbia University Vagelos College of Physicians and Surgeons, New York, New York, USA; Columbia University Vagelos College of Physicians and Surgeons, New York, New York, USA; Department of Medicine, Division of Infectious Diseases, Columbia University Irving Medical Center, New York, New York, USA; Department of Medicine, Division of Infectious Diseases, Columbia University Irving Medical Center, New York, New York, USA; Department of Medicine, Division of Infectious Diseases, Columbia University Irving Medical Center, New York, New York, USA; HIV Prevention Program in the Comprehensive Health Program, Ambulatory Care Network West, NewYork-Presbyterian Hospital, New York, New York, USA; Department of Pediatrics, Columbia University Irving Medical Center, New York, New York, USA; Department of Medicine, Division of Infectious Diseases, Columbia University Irving Medical Center, New York, New York, USA; HIV Prevention Program in the Comprehensive Health Program, Ambulatory Care Network West, NewYork-Presbyterian Hospital, New York, New York, USA; Columbia University Vagelos College of Physicians and Surgeons, New York, New York, USA; Department of Medicine, Division of Infectious Diseases, Columbia University Irving Medical Center, New York, New York, USA; HIV Prevention Program in the Comprehensive Health Program, Ambulatory Care Network West, NewYork-Presbyterian Hospital, New York, New York, USA; Columbia University Vagelos College of Physicians and Surgeons, New York, New York, USA

**Keywords:** ending the HIV epidemic, HIV Pre-exposure prophylaxis (HIV PrEP), provider feedback, sexually transmitted infections, sexual health

## Abstract

**Introduction:**

HIV preexposure prophylaxis (PrEP), a key strategy for preventing HIV transmission, requires awareness and access to PrEP services. Although all patients should be made aware of HIV PrEP; the diagnosis of bacterial sexually transmitted infections (STIs) is an important indicator of potential HIV PrEP need. In a previous evaluation of Get2PrEP (G2P), we found that an electronic medical record laboratory comments did not increase the frequency of PrEP discussions between patients and providers. In Get2PrEP2 (G2P2), we hypothesized that active, personalized messaging to providers about HIV PrEP would increase the documentation of PrEP discussions, referrals, and/or provision of HIV PrEP to individuals diagnosed with an STI.

**Methods:**

G2P2 was a parallel 3-arm, unblinded, randomized controlled design. Participants were allocated 2:1 to intervention or control. Participants in the intervention arm were further allocated to receive provider messaging through the electronic medical record chat message or e-mail.

**Results:**

The 191 randomized encounters resulted in a modest 7.8% (odds ratio, 1.078; confidence interval, 1.02–1.13) increase in documented PrEP discussions in intervention encounters versus none in the standard care group. There was no statistical difference by intervention modality. All documented discussions occurred in the outpatient or emergency department and were more frequent in women and those aged <25 years.

**Discussion:**

An e-mail or electronic medical record chat message sent to providers of patients testing positive for an STI had a small but significant effect on documented patient-provider PrEP discussions. Further investigation is required to determine whether provider messaging can increase PrEP uptake among eligible patients and longer-term outcomes.

## Teaser

An e-mail or electronic medical record chat message sent to providers of patients testing positive for a sexually transmitted infection increased documented patient-provider PrEP discussions (odds ratio, 1.078; confidence interval, 1.02–1.13) within a short period.

HIV preexposure prophylaxis (PrEP) is a highly effective intervention for preventing HIV acquisition and a key strategy to ending the HIV epidemic [1]. However, to fully benefit from this intervention, patients must be aware of HIV PrEP, linked to services with an HIV PrEP provider, and willing and able to use HIV PrEP effectively [2–4]. The Centers for Disease Control and Prevention currently recommends that all sexually active adults and adolescents be informed about HIV PrEP [5]. Although all persons should be informed about HIV PrEP, indicators for PrEP use have been elusive. The diagnosis of bacterial sexually transmitted infections (STIs) is an important indicator of potential HIV PrEP need. In New York City, historically, an STI diagnosis has been associated with increased vulnerability to HIV acquisition [6–8]. By identifying individuals with a higher likelihood of HIV acquisition through STI screening, health care systems can develop interventions to increase the offer of PrEP as a preventive measure.

New York (Manhattan) and Bronx counties represent 2 of the ending the HIV epidemic jurisdictions, a collection of 50 locales that account for more than half of new HIV diagnoses across the United States [9]. The Comprehensive Health Program (CHP) at Columbia University Irving Medical Center—NewYork Presbyterian Hospital serves Northern Manhattan and the Bronx communities disproportionately affected by the syndemic of HIV and STIs. CHP has clinical navigators who discuss HIV PrEP with all patients without HIV at their first sexual health visit and document the outcome of that discussion. Prior studies in this setting demonstrated a significant gap in the provision of PrEP to individuals diagnosed with an STI outside of dedicated sexual health settings. Specifically, we found that only 5% of individuals with an STI diagnosed outside of our sexual health program had a documented discussion about PrEP with their health care providers. Moreover, this discrepancy was even more significant for women than men, with only 1.1% of women having discussions about PrEP compared to 17% of men [10]. These findings suggest that there is a critical need for interventions to increase awareness and uptake of PrEP among individuals vulnerable to HIV acquisition, particularly women. In nonsexual health settings, linking the diagnosis of bacterial STIs with PrEP is a promising strategy to increase its uptake.

Studies have shown that passive interventions, such as interpretive laboratory comments, can effectively reduce inappropriate antibiotic prescribing [11–13]. Building on these findings, in our first Get2PrEP project, we investigated whether adding an electronic medical record (EMR) laboratory comment with PrEP and program contact information to all positive STI results would increase the frequency of PrEP discussions and prescriptions between patients and providers and narrow the gap between men and women. Get2PrEP did not impact PrEP discussions or disparities as hypothesized [14]. The frequency of PrEP discussions between patients and providers did not differ, and the gap between men and women remained [14]. These results suggest that additional more active strategies are needed to increase the uptake of PrEP among individuals diagnosed with STIs.

Prospective audits with provider feedback have been shown to be an effective strategy for modifying provider behavior in health care settings. This strategy can optimize and reduce antibiotic use and is a core tenet of almost all hospital antimicrobial stewardship programs [15]. It has also been shown to reduce the prevalence of uncorrected HIV prescribing errors at discharge [15]. At our institution, a provider feedback system that informed providers monthly about their individual HIV and hepatitis C testing rates resulted in notable increases in HIV and hepatitis C virus testing [16].

Therefore, in the Get2PrEP2 project, we aimed to increase the awareness, uptake, and use of HIV PrEP among individuals testing positive for an STI in clinical services outside of sexual health using active messaging. We hypothesized that active, personalized messaging to providers about HIV PrEP would increase the documentation of PrEP discussions, referrals, and/or provision of HIV PrEP in individuals diagnosed with an STI outside of dedicated sexual health settings.

## METHODS

### Study Design

This pilot quality improvement project was a parallel 3-arm, unblinded, randomized controlled trial. Participants were allocated 2:1 to intervention or control, with participants in the intervention arm being further allocated to provider messaging through the EMR or e-mail. We conducted the project adhering to the CONSORT statement for randomized trials of nonpharmacologic treatments [17].

### Sampling Frame and Eligibility

Between 28 June and 10 September 2021, we used a sexual health dashboard to identify potentially eligible encounters on each business day (Monday through Friday). The dashboard identified all tests ordered for gonorrhea, chlamydia, syphilis, and HIV across Columbia University Irving Medical Center—NewYork Presbyterian Hospital, which included outpatient, emergency department (ED), and inpatient testing. Clinical encounters were manually reviewed for PrEP eligibility.

Encounters were considered eligible if they (1) included a patient who had tested positive for gonorrhea, chlamydia, or syphilis at that visit; (2) tests were ordered from any clinical setting outside of 2 clinics that routinely provide sexual health and HIV prevention services at the institution; and (3) there was no documentation of any provider discussing or prescribing PrEP in the 12 months before the encounter date. Although gonorrhea and chlamydia were defined by the reported result, a positive syphilis test was defined using the traditional algorithm and manual review; for cases in which positivity remained unclear, we used the provider's documented interpretation.

Encounters were ineligible if conducted in the sexual health program, PrEP had been discussed or prescribed during the clinical visit, the patient had HIV, or the encounter represented a repeat positive test result within the previous month. We also excluded encounters in which patients had concurrent medical illnesses severe enough that PrEP discussion would not have been appropriate.

### Intervention

Eligible encounters were randomized to 1 of the 3 arms: (1) standard of care, (2) standard of care plus provider outreach through an email message, or (3) standard of care plus provider outreach through EMR chat message. Provider messaging consisted of a notification regarding STI diagnosis and a prompt to counsel on PrEP. Because of the nature of the intervention, data abstractors could not be blinded to the intervention arm of the encounter.

#### Arm 1

Standard of care meant that each clinical site would follow their local process for returning STI results, follow-up, and treatment. There is no medical center-wide standard process for identifying these patients to ensure they are treated or linked to care. The standard of care included the institution's existing CHP, which routinely provides provider education and outreach throughout the institution to increase awareness about available services. Providers had access to the program's information through routine outreach efforts, ongoing advertising, and the interpretive laboratory comment posted below every positive gonorrhea and chlamydia test result in the EMR [14]. CHP has 5 sexual health navigators available via a phone warm-line (9 Am–5 Pm) to provide virtual care navigation and assist with linkage to care.

#### Arms 2 and 3

After identifying an eligible encounter, a provider from our sexual health team sent a message (by e-mail in arm 2 vs through the EMR chat in arm 3) to the diagnosing provider within 3 business days of ordering an STI test that was positive (Supplementary Text). Regardless of the delivery method, the content of the provider messages was standardized and identical. Although providers did not need to respond, they could consult with the sexual health prevention team.

### Power and Sample Size Estimation

We conducted a power analysis for a 2-tailed hypothesis test, aiming to detect a 3-fold increase in the primary outcome of documentation of PrEP counseling or prescription from 7% in the control group to 25% in the intervention group. With an alpha level of 0.05 and a desired power of 80%, our calculations indicated that 98 participants in the intervention arm and 49 participants would be required in the control arm, assuming a 2:1 randomization ratio.

### Randomization

A randomization scheme was developed for a minimum of 147 encounters based on the sample size estimation. Randomization was 1 to 1 to 1 and performed using the “=rand()” function in Excel. The distribution was broken into thirds, representing each arm. Enrolled patients were consecutively assigned a randomization value and the corresponding arm.

### Outcomes

Outcomes were assessed through a manual electronic medical record review 4 weeks after the encounter date. The primary outcome was a composite of the following documented HIV PrEP services: (1) referrals to the HIV prevention program, (2) prescription for HIV PrEP, or (3) mention of “HIV PrEP,” “PrEP,” “preexposure prophylaxis” in clinical documentation.

Secondary outcomes include documentation of other sexual health counseling indicated by the terms; “safe sex,” “condoms,” or “postexposure prophylaxis.” Safe sex was defined as any form of STI prevention education, whereas condoms included recommending or providing condoms and other forms of barrier protection. This is consistent with prior studies [14]. It is important to acknowledge that sexual health is moving away from ambiguous, imprecise, and stigmatizing language such as “unsafe” sex or “risky” sex; however, many providers still use the terms and, therefore, we chose to include them in our data analysis [18].

### Statistical Analysis

We summarized the baseline demographic and clinical characteristics of the patients involved in the encounters, including STI testing and results at the encounter, reasons for STI testing, behaviors associated with HIV and/or STI acquisition, and receipt of HIV prevention and/or PrEP services. Baseline characteristics were summarized for the entire sample by arm. We used chi-square tests of independence and analysis of variance to assess imbalance in arms by baseline categorical and continuous variables, respectively. Because of our small number of events, we conducted descriptive univariate and bivariate analyses only. We calculated the Fisher exact to test the difference between the intervention (arms 2 and 3) and standard of care arm and computed the Cochran-Mantel-Hazel odds ratio for the magnitude of effect and 95% confidence intervals (CI). Statistical significance was determined as a *P* <.05. Because we detected a difference, we compared the frequency of the outcomes between the 3 arms also using the Fisher exact test. We assessed the level of missingness for all baseline characteristics. We stratified by sex to explore possible differences. Data were analyzed using R Studio 4.3.1 (package) and SAS version 9.4.

### Additional Exploratory Analyses

We performed an analysis of secondary outcomes to evaluate for changes in the documentation of other HIV prevention topics, including “safe sex,” “condoms,” and “Post-exposure prophylaxis (PEP).”

### Patient Consent Statement

This project was approved by the Columbia University institutional review board with a waiver of informed consent.

## RESULTS

### Included Population

**Figure 1.**
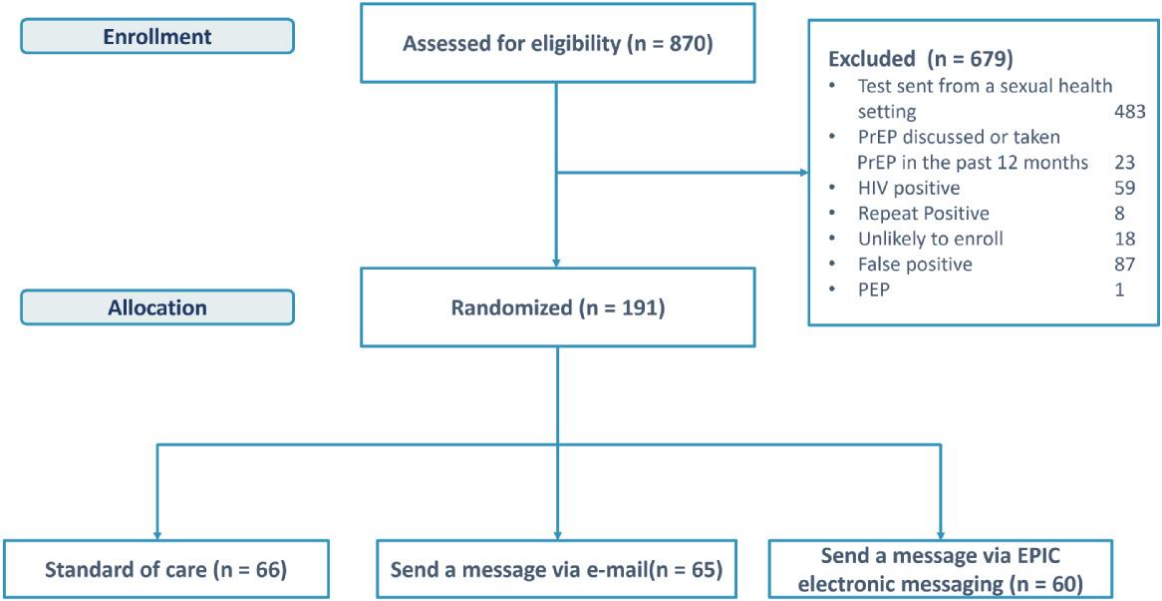
Population randomization schema.

A total of 870 encounters with positive STI laboratory results were identified during the project period; 679 were excluded, most frequently because STI testing for those encounters was performed in CHP. Exactly 191 encounters were randomized: 66 to standard of care, 65 to provider e-mail message, and 60 to provider EMR message (Figure 1).

Patients were aged 14 to 24 years (58%), mostly cisgender women (78%), and self-identified as Hispanic (62%), non-Hispanic Black (18%), or non-Hispanic White (4.2%). Most patients were seen and tested in the outpatient setting (50%) or the ED (48%). A total of 28% of patients were pregnant. Chlamydia was the most commonly diagnosed STI (151 participants, 81%), followed by syphilis (16 participants, 19%) and gonorrhea (33 participants, 18%). Most patients had no documented behaviors associated with STI acquisition (Table 1). The most common reasons for STI testing included routine care (39%), symptoms (35%), routine STI care (18%), and sexual encounter (8.9%). Among symptomatic patients, the most common symptoms were discharge (19%), dysuria (19%), and abdominal pain (18%) (Supplementary Table 1).

**Table 1.** Baseline Characteristics of Participants by Randomized Arm

	Overall n (%)	Standard of Care N = 66	Intervention N = 125
Age, y			
14–24	110 (58%)	36 (55%)	74 (59%)
25–34	58 (30%)	19 (29%)	39 (31%)
>34	23 (12%)	11 (17%)	12 (10%)
Gender			
Cis-male	148 (78%)	54 (82%)	94 (76%)
Cis-female	41 (22%)	12 (18%)	29 (23%)
Other	1 (0.5%)	0 (0%)	1 (0.8%)
Race/ethnicity			
Hispanic	118 (62%)	37 (56%)	81 (65%)
Non-Hispanic Black	35 (18%)	9 (14%)	26 (21%)
Non-Hispanic White	8 (4.2%)	4 (6%)	4 (3.2%)
Other	29 (15%)	16 (24%)	13 (10%)
Unknown	1 (0.5%)	0 (0%)	1 (0.8%)
Care location			
Inpatient	3 (1.6%)	2 (3.0%)	1 (0.8%)
Outpatient	96 (50%)	34 (52%)	62 (50%)
Emergency department	92 (48%)	30 (45%)	62 (50%)
Pregnant (yes)	36 (28%)	10 (21%)	26 (32%)
STI results			
Chlamydia positive	151 (81%)	50 (79%)	101 (82%)
Gonorrhea positive	33 (18%)	12 (19%)	21 (17%)
Syphilis positive	16 (19%)	6 (18%)	10 (19%)
STI risk factors^a^			
MSM	9 (4.8%)	2 (3.1%)	7 (5.6%)
History of IVDU	2 (1.1%)	1 (1.5%)	1 (0.8%)
Partner with HIV	1 (0.5%)	0 (0%)	1 (0.8%)
None documented	177 (94%)	62 (95%)	115 (93%)

Abbreviations: IVDU, intravenous drug use; MSM, men having sex with men; STI, sexually transmitted infection.

^a^Condomless sex excluded because of poor documentation.

All patients received genitourinary STI testing, 3% received pharyngeal testing, and 2.6% received rectal testing. Forty-five percent (N = 86) of patients were concurrently tested for syphilis, 45% (85) of patients were concurrently tested for HIV with no reactive results (Supplementary Table 2), and 87% (166) of patients had documented treatment within 4 weeks of their STI diagnosis.

## IMPACT ON PREP DISCUSSIONS AND OTHER HIV PREVENTION DISCUSSIONS

**Figure 2.**
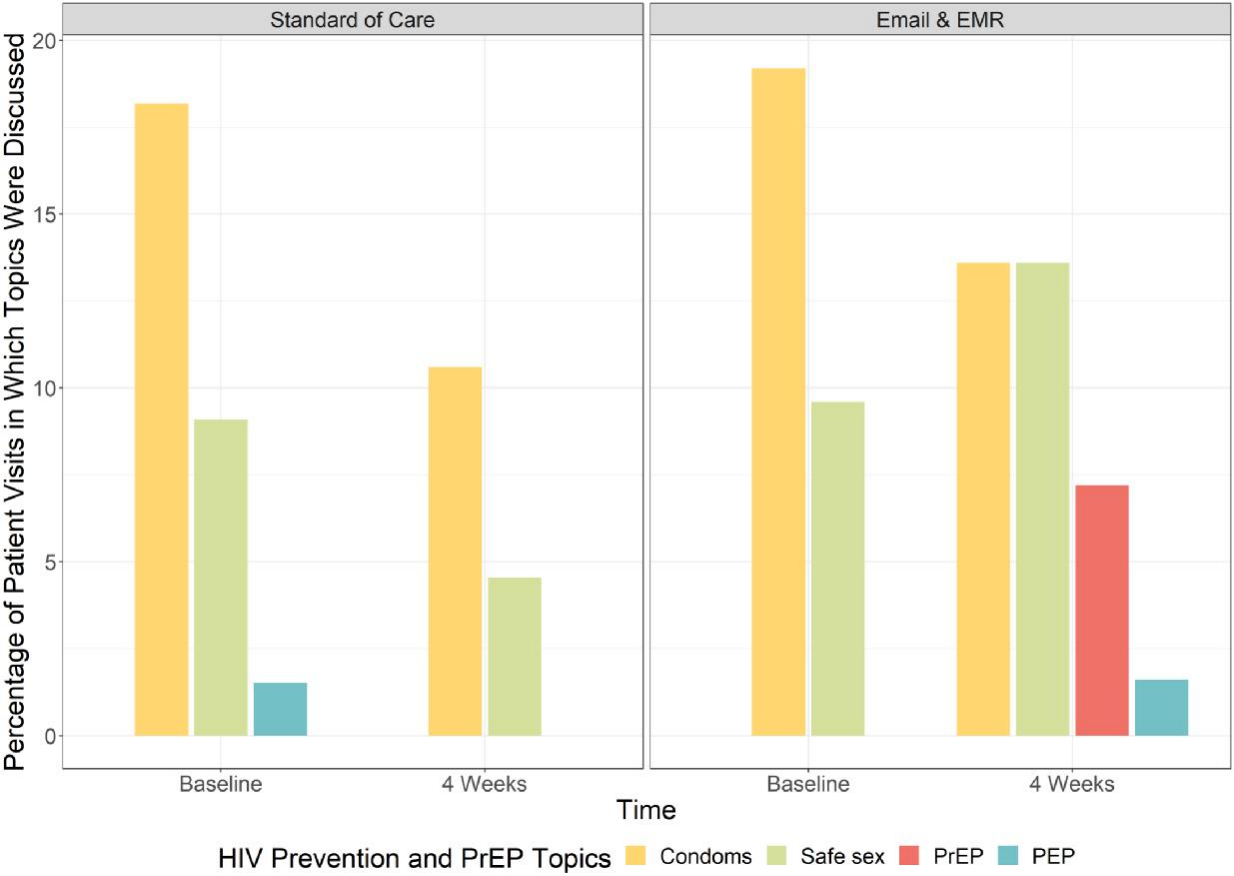
Provider documentation of HIV PrEP discussion and other sexual health topics among individuals by randomization.

**Figure 3.**
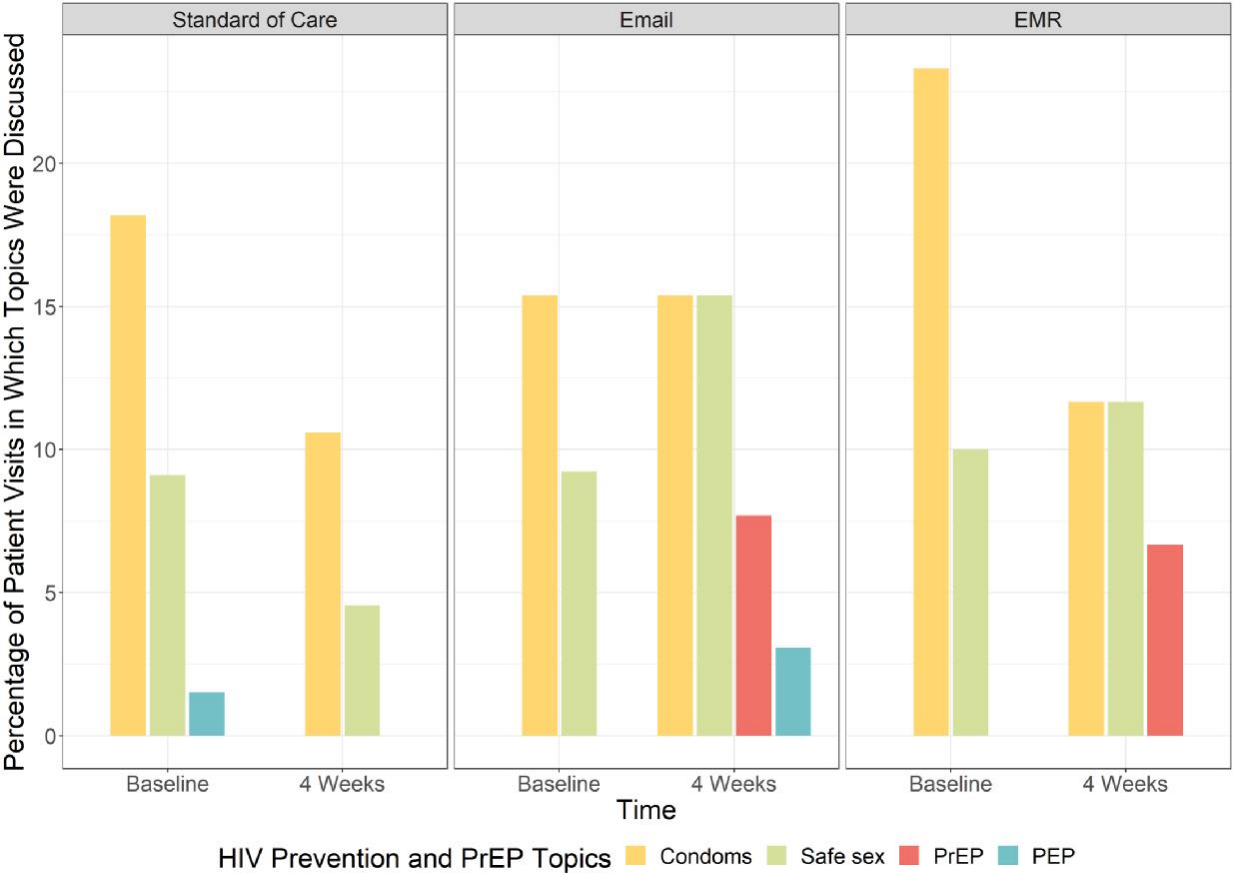
Provider documentation of HIV PrEP discussion and other sexual health topics among individuals by messaging method.

**Figure 4.**
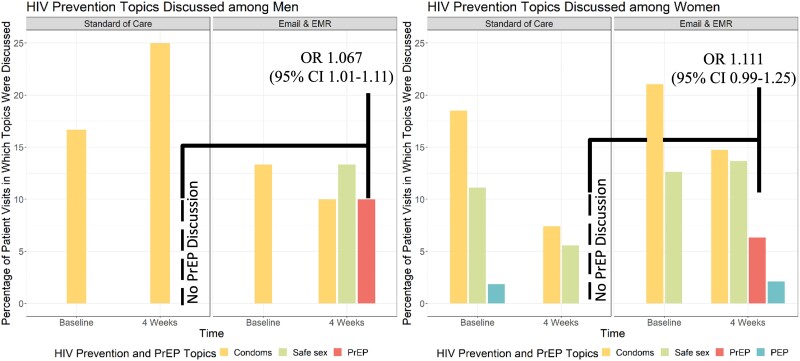
Provider documentation of HIV PrEP discussion and other sexual health topics among individuals by sex assigned at birth.

The primary outcome was planned as the composite outcome of PrEP discussions, referrals, and prescription among patients with eligible encounters. However, no patients had evidence of a referral or PrEP prescription within 4 weeks of their STI diagnosis. In the intervention arms, 7.2% had documented PrEP discussions, compared to 0% in the standard of care arm. Patients whose providers received any message (e-mail or EMR) were 7.8% (odds ratio [OR], 1.078; 95% CI, 1.02–1.13) more likely to document PrEP discussion (Figure 2). PrEP discussion did not differ statistically between e-mail messages (7.7%) and EMR messages (6.7%; *P* = .26) (Figure 3). All documented PrEP discussions occurred among patients age ≤24 years. PrEP discussions did not differ significantly by any other demographic or STI history variables. No PrEP discussions were documented in the inpatient setting compared to the outpatient (5%) and ED (4.2%) settings (Table 2). When stratified by self-identified gender, cis-females whose providers received a message were 6.7% (OR, 1.067; 95% CI, 1.01–1.12) more likely to have PrEP discussed than standard of care. When stratified by age (≤24 vs >24 years), adolescents and young adults age ≤24 years whose providers received a message were more likely to have a PrEP discussion documented than those >24 years (OR, 1.08; 95% CI, 1.02–1.15; *P* = .01). Cis-males had an 11% (OR, 1.111; 95% CI, .99–1.25) increase in PrEP discussions than cis-males in standard of care, though the CIs included the null (Figure 4).

**Table 2.** Baseline Characteristics of Participants by PrEP Discussion Outcome

Characteristic	N	Overall N = 199	PrEP Discussed N = 9	PrEP Not Discussed N = 190
Age, y	199	…	…	…
14–24	…	116 (58%)	9 (100%)	107 (56%)
25–34	…	60 (30%)	0 (0%)	60 (32%)
>34	…	23 (12%)	0 (0%)	23 (12%)
Gender	198	…	…	…
Ciswoman	…	156 (79%)	6 (67%)	150 (79%)
Cisman	…	41 (21%)	3 (33%)	38 (20%)
Other	…	1 (0.5%)	0 (0%)	1 (0.5%)
Race/ethnicity	198	…	…	…
Hispanic	…	126 (64%)	8 (89%)	118 (62%)
Non-Hispanic Black	…	35 (18%)	0 (0%)	35 (19%)
Non-Hispanic White	…	8 (4.0%)	1 (11%)	7 (3.7%)
Other	…	29 (15%)	0 (0%)	29 (15%)
Patient status	199	…	…	…
Inpatient	…	3 (1.5%)	0 (0%)	3 (1.6%)
Outpatient	…	102 (51%)	5 (56%)	97 (51%)
ED	…	94 (47%)	4 (44%)	90 (47%)
Pregnant at the time of laboratory test	137	…	…	…
Yes	…	42 (31%)	2 (40%)	40 (30%)
No	…	95 (69%)	3 (60%)	92 (70%)
Chlamydia positive	194	157 (81%)	6 (67%)	151 (82%)
Specimen tested positive for CT	157	…	…	…
Pharyngeal	…	0 (0%)	0 (0%)	0 (0%)
Rectal	…	2 (1.3%)	0 (0%)	2 (1.3%)
GU	…	155 (99%)	6 (100%)	149 (99%)
Other	…	0 (0%)	0 (0%)	0 (0%)
Gonorrhea positive	194	35 (18%)	3 (33%)	32 (17%)
Location of positive gonorrhea	35	…	…	…
Pharyngeal	…	3 (8.6%)	0 (0%)	3 (9.4%)
Rectal	…	0 (0%)	0 (0%)	0 (0%)
GU	…	32 (91%)	3 (100%)	29 (91%)
Other	…	0 (0%)	0 (0%)	0 (0%)
Syphilis test result	90	…	…	…
Reactive	…	18 (20%)	1 (25%)	17 (20%)
Nonreactive	…	72 (80%)	3 (75%)	69 (80%)

Abbreviations: CT, chlamydia trachomatis; ED, emergency department; GU, genitourinary; PrEP, preexposure prophylaxis.

## DISCUSSION

Our quality improvement project aimed to assess the impact of personalized provider messaging on documentation of PrEP discussions within 4 weeks of patient-provider encounters with a new STI diagnosis. Providers who received a message were more likely to document PrEP discussion overall. There was not a statistically significant difference between the 2 messaging modalities. Personalized provider messaging improved documentation for females and males, although the 95% CI included the null for males and was effective in adolescents and young adults (age ≤24 years). Assuming that PrEP documentation reflects patient-provider discussion, these findings suggest that provider messaging may influence PrEP discussions in the near term. Further, documentation of PrEP discussion in vulnerable populations that have not historically benefitted from HIV-PrEP messaging, such as females, Hispanics, adolescents, and young adults, suggests the approach could support closing the gap in some disparities [19–21].

Various studies have shown that provider alerts can successfully increase screening for HIV and other infections. The downstream outcomes such as HIV diagnoses and PrEP prescriptions have been met with variable success [22–27]. In the ED at our institution, monthly individualized provider feedback about testing rates was extremely effective in increasing HIV and hepatitis C virus testing [16]. Providers may feel more comfortable with HIV/hepatitis C virus testing as an established part of routine care. Conversely, PrEP may be less familiar, attributed to the observation of no PrEP outcomes in the standard of care arm. Additionally, the low levels of PrEP screening and referral may be attributable to providers' perception that PrEP counseling and referral may be more complex than testing. HIV testing is a relatively straightforward, 1-time activity requiring brief pre-/postcounseling, an order, and patient notification. Providers may perceive PrEP as involving a more complex set of steps, in-depth counseling, medication initiation, and ongoing monitoring. Previous qualitative interviews and surveys of providers in this setting revealed PrEP barriers, including lack of knowledge and training, competing priorities, and time/resource constraints during clinical visits [28–30]. We further hypothesized that a personal provider message may overcome these barriers as the messages allowed for 2-way communication between the provider and the HIV prevention program.

In other settings, Ridgway et al. used predictive models to identify HIV PrEP candidates, which resulted in PrEP prescriptions for 7.8% of identified participants at risk [27]. Another EHR-based intervention guided by an HIV risk prediction model substantially increased the initiation of PrEP care but only among patients of primary care providers who also care for people with HIV [31]. Although provider education may seem like a solution to this issue, more than education may be needed as a recent enhanced provider training module did not significantly increase the number of participants prescribing HIV-PrEP [32]. A more thorough understanding of the types of training needed can help guide future efforts to improve PrEP uptake and delivery, such as tailored local educational resources and implementing system-level interventions such as trained navigators to support providers in PrEP discussions and initiation in diverse settings [33, 34]. A recent systematic review of PrEP opportunities in US EDs showed the feasibility of counseling, scheduling, and referring patients for PrEP care [35].

Further research is needed to understand institutional-level barriers and facilitators to provider messaging for PrEP outcomes. For instance, because of personalized messages to providers and regulatory limitations, we did not provide global feedback on PrEP discussions, but that could be a future strategy. Conversely, although HIV-PrEP discussions should be offered to all patients, including before results being known, in this study, we focused on individuals who had tested positive for an STI after the visit. This may have limited effectiveness in the ED, where providers also have limited interactions with patients after their initial visit. The messaging modalities used allowed for easier adding of other providers to the chat, which, although not formally captured, was anecdotally noted to happen frequently. A strategy of reaching out to patients' primary care doctors as opposed to the ED or subspecialists offers another potential strategy. Finally, given the barriers of time and complexity of PrEP discussions, a future strategy a direct patient messaging strategy from the HIV prevention team (since renamed the sexual health team) to the patient would guarantee the patient had some information about PrEP and may facilitate uptake.

Several aspects of this project strengthened the internal validity. Randomization was balanced for patient characteristics. Conceivably, patients and providers could have been represented in multiple encounters. The inclusion/exclusion criteria for patients reduced the likelihood of patients having multiple encounters because the project period was short, 3 months, and encounters for repeated STIs within 1 month were ineligible. Providers could have repeated encounters. Although we could not examine the provider effect, we examined service location. Our selection and randomization established a clear temporal relationship between the alert and counseling. Further, the short observation period reduced time-dependent biases that may arise, but also may explain why we observed so few events. Also, the sample was representative of the population vulnerable to STI and HIV and experiencing disparity; they were mainly young, cisgender women who self-identified as Hispanic, non-Hispanic White, or non-Hispanic Black. Most patients were seen and tested in the outpatient setting or ED, with a substantial proportion of pregnant patients. These demographic and clinical characteristics are consistent with the population most vulnerable to STIs and to some extent, HIV [36]. Concerningly, this is also the population least likely to be started on PrEP, highlighting the importance of improving access to PrEP outside of the sexual health setting [37].

This project had several limitations. First, we did not observe enough events to conduct regression analyses and control for potentially confounding variables of the intervention effect. As previously noted, our observation time could have been extended to observe more events, with further design considerations to maintain internal validity. We hypothesized a 7% event rate in the standard of care arm and observed 0%, which posed the greatest statistical challenge. Second, as noted previously, potential clustering by service location, provider, or patient may have skewed events or association, and we could not fully describe and control at all levels. Future studies can address this. Third, several measurement factors could have affected the observed association. Our observation window for the PrEP outcome may have been too narrow. The following clinical visit when PrEP could likely be discussed may be long and variable and the provider may not have seen the e-mail or note in time for their patient encounter. We did not assess for how “many” visits patients had after their diagnosis or ensure that all patients had at least 1 visit. Measuring at 3 to 6 months after the intervention may have identified more visits and potentially more discussions and could be examined in another project. Further investigation is required to determine whether provider messaging can increase PrEP uptake among eligible patients as well as longer term outcomes.

Overall, our quality improvement project showed that a provider alert impacted patient-provider PrEP discussions, and, interestingly, for females where there are few interventions with evidence in the United States. Our findings can hopefully help inform the development of future interventions as part of ending-the-HIV epidemic efforts.

## Supplementary Material

ofae297_Supplementary_Data

## Supplementary Data

Supplementary materials are available at the *Journal of The Pediatric Infectious Diseases Society* online (http://jpids.oxfordjournals.org). Supplementary materials consist of data provided by the author that are published to benefit the reader. The posted materials are not copyedited. The contents of all supplementary data are the sole responsibility of the authors. Questions or messages regarding errors should be addressed to the author.

## Notes

***Author contributions.*** J. Z. and D. C. contributed equally to this manuscript. J. Z., D. C., D. T., C. C., K. M., A. C., P. G., and M. S. conceived the project and its design. J. Z., D. C., and S. H. had full access to the data and take responsibility for the integrity of the data and accuracy of the analysis. E. O., N. A., L. B., E. W., N. M., D. T., C. C., and J. Z. organized and entered data. J. S. and D. C. drafted the manuscript. All authors contributed to data interpretation and have read and agreed to the published version of the manuscript.

***Data availability.*** Data not publicly available.

***Financial support*.** This research was supported by the HIV Vaccine Trials Network Research and Mentorship Program (E. O., N. A., L. B.). Research reported in this publication was also supported by the National Institute of Allergy and Infectious Diseases of the National Institutes of Health under award numbers UM1AI069470 (J. Z., D. T., M. E. S.), K23AI150378 (J. Z.), and L30AI133789 (J. Z.).
