# Supplementary material for: Get2PrEP2—A Provider Messaging Strategy to Improve PrEP Uptake: A Randomized Controlled Quality Improvement Project
Source: Open Forum Infect Dis. 2024 Jun 20;11(6):ofae297. doi: 10.1093/ofid/ofae297 (PMC11191360; doi:10.1093/ofid/ofae297)
Supplement: ofae297_Supplementary_Data [file ofae297_supplementary_data.docx]

**Supplemental Text 1: Standardized Provider E-mail Message**

#Encrypt Subject: Message from HIV Prevention Team regarding your patient with a recent positive STI test

Good afternoon [Physician Name],

I am a member of the HIV Prevention Team at NYP/CUIMC and we were alerted about a positive [gonorrhea, chlamydia, or syphilis test] for your patient [MRN: XXXXXXX].

Based on this positive test, your patient may meet CDC and/or DOH criteria for HIV pre-exposure prophylaxis (PrEP). Consider sending an HIV test if you have not already done so and discussing PrEP.

The HIV Prevention Program is available to provide further information and/or assistance with linkage to HIV prevention care. *If you have a patient, you think might benefit from our services please ask for permission to have our team contact them.*  While we cannot provide patient results, we are happy to reach out to patients to provide education about HIV prevention services and linkage to ongoing sexual health and HIV prevention services.

We are reachable Monday – Friday between 9 am -5pm at (973) 475-8498; you can also e-mail us at [HIVPREVENTION@NYP.ORG](mailto:HIVPREVENTION@NYP.ORG) for a response within 24 hours.

For additional information and learning modules on STI testing, treatment or PrEP, or to learn more about our program, please visit [http://www.stick2prep.com](http://www.stick2prep.com/)

Please note that this message is part of a quality improvement effort from the HIV prevention team to encourage providers to identify and refer patients who might benefit from HIV prevention services. While this e-mail was about a particular case, if your relationship with the patient has ended, we hope that this information and resources may be beneficial to you for future patients.

Please let us know if we can provide any other assistance regarding STI testing, treatment, and prevention.

Thank you for your time,

[Person Sending E-mail]

Supplemental Table 1: Indications for STI Testing

| **Symptoms*** | **Overall**    **n (%)** | **Standard of Care**   **N=66** | **E-mail Message**   **N=65** | **EPIC Message**   **N=60** | **p-value** |
| --- | --- | --- | --- | --- | --- |
| Discharge (GU) | 37 (19%) | 9 (14%) | 18 (28%) | 10 (17%) | 0.1 |
| Burning/Dysuria | 36 (19%) | 11 (17%) | 13 (20%) | 12 (20%) | 0.9 |
| Abdominal Pain | 41 (21%) | 11 (17%) | 21 (32%) | 9 (15%) | 0.031 |
| Rash | 10 (5.2%) | 2 (3.0%) | 3 (4.6%) | 5 (8.3%) | 0.4 |
| Vaginal Symptoms | 14 (7.3%) | 6 (9.1%) | 6 (9.2%) | 2 (3.3%) | 0.4 |
| Nausea and Vomiting | 14 (7.3%) | 5 (7.6%) | 5 (7.7%) | 4 (6.7%) | > 0.9 |
| Diarrhea | 6 (3.1%) | 2 (3.0%) | 4 (6.2%) | 0 (0%) | 0.15 |
| Hematuria | 5 (2.6%) | 1 (1.5%) | 2 (3.1%) | 2 (3.3%) | 0.7 |
| Pelvic Pain | 5 (2.6%) | 2 (3.0%) | 3 (4.6%) | 0 (0%) | 0.3 |
| PID | 5 (2.6%) | 3 (4.5%) | 2 (3.1%) | 0 (0%) | 0.4 |
| Sore Throat | 4 (2.1%) | 0 (0%) | 1 (1.5%) | 3 (5.0%) | 0.076 |
| Neuro Symptoms | 4 (2.1%) | 0 (0%) | 3 (4.6%) | 1 (1.7%) | 0.2 |
| Rectal Bleeding | 3 (1.6%) | 1 (1.5%) | 2 (3.1%) | 0 (0%) | 0.7 |
| Ocular Symptoms | 2 (1.0%) | 1 (1.5%) | 1 (1.5%) | 0 (0%) | >0.9 |
| Rectal Pain | 1 (0.5%) | 0 (0%) | 1 (1.5%) | 0 (0%) | 0.7 |
| Other** | 34 (18%) | 10 (15%) | 9 (14%) | 15 (25%) | 0.2 |
| No Symptom | 72 (38%) | 30 (45%) | 19 (29%) | 23 (38%) | 0.2 |
| Any Symptom | 119 (62%) | 36 (55%) | 46 (71%) | 37 (62%) | 0.2 |
| **Reasons for Testing** |  |  |  |  | 0.4 |
| Routine Care*** | 74 (39%) | 26 (39%) | 24 (37%) | 24 (40%) |  |
| Symptoms | 66 (35%) | 18 (27%) | 28 (43%) | 20 (33%) |  |
| Routine STI*** | 34 (18%) | 16 (24%) | 7 (11%) | 11 (18%) |  |
| Sexual Encounter | 17 (8.9%) | 6 (9.1%) | 6 (9.2%) | 5 (8.3%) |  |
| *Symptoms are not mutually exclusive  ** Symptoms with >2 responses not included: back pain, chest pain  ***Routine care included annual STI screening at a primary care visit vs routine STI care individuals were specifically asking for STI testing. | | | | | |

Supplemental Table 2: Sexually Transmitted Infection Positivity by Arm

|  | **Overall**    **n (%)** | **Standard of Care**   **N=66** | **E-mail Message**   **N=65** | **EPIC Message**   **N=60** |
| --- | --- | --- | --- | --- |
| **Genitourinary Testing**  (n=186) |  |  |  |  |
| Chlamydia | 150 (81%) | 49 (78%) | 53 (83%) | 48 (83%) |
| Gonorrhea | 30 (16%) | 12 (19%) | 11 (17%) | 7 (7%) |
| **Pharyngeal testing**  (n=6) |  |  |  |  |
| Chlamydia | 1 (17%) | 0 (0%) | 1 (33%) | 0 (0%) |
| Gonorrhea | 3 (50%) | 0 (0%) | 2 (66%) | 1 (100%) |
| **Rectal testing**  (N=5) |  |  |  |  |
| Chlamydia | 2 (40%) | 2 (40%) | 0 (0%) | 0 (0%) |
| Gonorrhea | 0 (0%) | 0 (0%) | 0 (0%) | 0 (0%) |
| **Syphilis^4^**  (N=84) | 16 (19%) | 6 (18%) | 5 (23%) | 5 (16%) |
| **HIV**  (n=85) | 86 (45%) | 33 (50%) | 27 (42%) | 26 (43%) |
|  | | | | |
